# LATERAL EPICONDYLE SLIDING OSTEOTOMY IN KNEE ARTHROPLASTIES WITH VALGUS DEFORMITY

**DOI:** 10.1590/1413-785220243201e268054

**Published:** 2024-05-06

**Authors:** Alessandro Rozim Zorzi, Bruno Suaed Foss, Pedro Henrique Calegari Moraes, Plínio de Almeida Martins de Souza, Gustavo Constantino de Campos, João Batista de Miranda

**Affiliations:** 1. Universidade Estadual de Campinas, Hospital de Clinicas, Departamento de Ortopedia, Reumatologia e Traumatologia, Campinas, SP, Brazil.; 2. Faculdade Sao Leopoldo Mandic, Disciplina de Ortopedia e Traumatologia, Campinas, SP, Brazil.

**Keywords:** Knee, Osteoarthritis, Arthroplasty, Genu Valgum, Osteotomy, Joelho, Osteoartrite, Artroplastia, Geno Valgo, Osteotomia

## Abstract

Objective: To evaluate the efficacy and safety of sliding osteotomy of the lateral epicondyle in correcting rigid valgus deformity in knee arthroplasty. Methods: A retrospective study of patients undergoing total knee arthroplasty with lateral epicondyle sliding osteotomy between 2006 and 2018. The main outcome was the incidence of complications and adverse events. Secondary outcomes were Visual Analog Scale for Pain, varus stress test, and varus knee thrust during gait. Results: 19 knees (19 participants) were included in the study. The mean follow-up was 4.2 years. There were no cases of infection or reoperation due to instability. Two participants (10.5%) had mild or moderate knee pain (VAS pain = 4.6 ± 1.9). Two arthroplasties (10.5%) had mild varus stress. No participant presented varus thrust. Conclusion: Sliding osteotomy of the lateral epicondyle allows fast and safe ligament balance of knee valgus deformities. ***Level of Evidence I, Case series.***

## RESUMO

*Avaliar a eficácia e a segurança da osteotomia de deslizamento do epicôndilo lateral na correção da deformidade em valgo rígida na artroplastia de joelho. Métodos: Estudo retrospectivo de pacientes submetidos à artroplastia total do joelho com osteotomia de deslizamento do epicôndilo lateral entre 2006 e 2018. O principal desfecho foi a incidência de complicações e eventos adversos. Os desfechos secundários foram escala visual analógica para dor, teste de estresse em varo e flambagem em varo do joelho durante a marcha. Resultados: Foram incluídos no estudo 19 joelhos (19 participantes). O seguimento médio foi de 4,2 anos. Não houve nenhum caso de infecção ou reoperação devido à instabilidade. Dois participantes (10,5%) apresentaram algum tipo de dor leve ou moderada no joelho (EVA = 4,6 ± 1,9). Duas artroplastias (10,5%) apresentaram estresse em varo leve. Nenhum participante apresentou flambagem em varo. Conclusão: A osteotomia de deslizamento do epicôndilo lateral possibilitou o balanço ligamentar das deformidades em valgo do joelho de forma rápida e segura.* ***Nível de Evidência IV, Série de Casos.***

## INTRODUCTION

 Valgus deformity of the knee can be defined as the presence of an angle between the anatomical axes of the femur and tibia equal to or greater than 10 degrees. Valgus accounts for only 10% of total knee arthroplasties (TKAs) and can represent a challenge for the surgeon. Although osteoarthritis is the most common pathology related to this deformity in adults, other inflammatory pathologies such as rheumatoid arthritis, systemic lupus erythematosus, psoriatic arthritis and hemophilic arthropathy are also associated. ^1^^,^^2^

 Adequate correction of the deformity in the coronal plane is widely accepted as crucial to the success of a TKA. ^3^ It is recognized that the correction of a valgus deformity has technical particularities that need to be recognized and addressed by the knee surgeon. It includes the access route, bone cuts and ligament balance. ^1^^,^^2^^,^^4^ Several ligament release techniques to correct coronal alignment in valgus knee arthroplasty have been described. ^1^^,^^5^^-^^7^ An elegant and efficient alternative is the sliding osteotomy of the lateral epicondyle described by Brilhault et al. ^8^ This osteotomy releases a bone block that contains the origins of both the lateral collateral ligament (LCL) and the popliteal tendon. The LCL is a crucial factor in knee valgus balance because it influences both extension and flexion space. Therefore, simply sectioning this structure may not achieve a good balance between the two spaces. Repositioning the epicondyle, on the other hand, allows the surgeon to better equalize these spaces. However, the original description of the surgical technique of this osteotomy is laborious. 

This study aims to evaluate the safety and effectiveness of performing a sliding osteotomy of the lateral epicondyle with a simplified technique, without the need to create a square around the epicondyle and without fixing the osteotomy with a screw.

## MATERIALS AND METHODS

This study was approved by the local Research Ethics Committee (CAAE 16612719.6.0000.5404) and all selected individuals agreed to participate and signed the Informed Consent Form. This is an observational, retrospective, case series type clinical study. Records of all TKAs performed at a teaching hospital between 2006 and 2018 were retrieved.

The inclusion criteria were participants of both sexes, over 18 years of age, with severe primary or secondary osteoarthritis of the knee and valgus deformity, undergoing TKA with sliding osteotomy of the lateral femoral epicondyle and at least one year of post-operative follow-up.

The exclusion criteria were absence of clinical or radiographic data in the medical records and refusal to participate or sign the Informed Consent Form (ICF).

### Indication for sliding osteotomy of the lateral epicondyle

 Sliding osteotomy is indicated in fixed valgus deformity when, after release of the ilitibial band and bone cuts, the extension and flexion spaces still have a trapezoidal shape due to excessive lateral tension. ^8^

### Surgical technique

**Figure 1.**
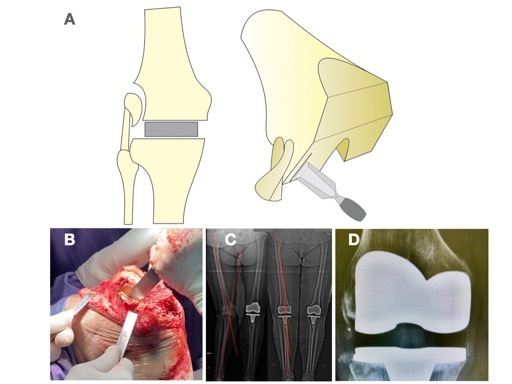
Modified surgical technique for performing sliding osteotomy of the lateral epicondyle in knee arthroplasties with irreducible valgus deformity.

 After delicate dissection and identification of the origins of the popliteal tendon and LCL, an incision is made in the periosteum in the distal region of the lateral condyle. A chisel Straight Lambotte is used to perform the lateral cortical osteotomy containing the insertion of the popliteus and lateral epicondyle originating from the LCL ( Figure 1 ). 

 A variation of the technique was used in all cases. There is no need to perform a square osteotomy around the fragment as per the classic description by Brilhault et al. ^8^ The advantage of this modification is to simplify the surgical procedure and maintain the periosteal insertion of the fragment, preserving its blood supply and ensuring relative stability. For this reason, at the end of the procedure, it is not necessary to fix the osteotomy with a screw. The fragment will accommodate itself in the best position according to the movement of the knee post-operatively. As part of the periosteum remains intact, the fragment still maintains some tension on the LCL. 

In the present study, the implant used in all cases was the cemented primary prosthesis Modular III (Víncula, Rio Claro – Brazil). The posterior cruciate ligament was sacrificed in all cases. All prostheses were cemented and the patellar component was used in all cases. No drain was used in any case.

### Clinical assessment

Information on the range of motion (ROM), visual analogue scale (VAS), presence of varus thrust during gait, varus stress test of the knee in full extension and at 30 degrees of flexion. The VAS scale was stratified into mild pain (1 to 3), moderate pain (4 to 6), severe pain (7 to 9) and unbearable pain (10). The varus stress test was classified as mild, moderate or severe subjectively by the examiner. The main outcome of this study was the incidence of adverse effects, mainly side pain, instability and revision surgery.

### Radiographic assessment

 Radiographs taken pre-operatively and during the post-operative follow-up were consulted. Krackow’s classification of knee valgus was used. ^9^^,^^10^ The anatomical femoraltibial (FT) angle was measured on standard monopodal radiographs in the frontal plane. 

### Statistical analysis

There was no prior calculation of the sample size as it was a convenience sample. Qualitative variables appear as absolute and relative frequencies. Quantitative variables appear as mean, standard deviation (SD) and range.

The distribution of each continuous variable was assessed by the Kolmogorov-Smirnov test. Mean results were compared with Student’s t-tests for independent or paired samples or the Mann-Whitney test for independent samples and the Wilcoxon test for paired samples when these assumptions were rejected. Pearson’s chi-squared test or Fisher’s exact test were used for comparisons of qualitative variables. All tests were two-tailed. The significance level was set at 5% (p < 0.05). Analyses were carried out using SPSS version 22.0 (Armonk, NY: IBM Corp.).

## RESULTS

Between 2006 and 2018, 52 patients who underwent total knee arthroplasty for valgus deformity were identified, 19 of whom underwent sliding osteotomy of the lateral epicondyle.

Table 1 presents patient demographic data. There was a predominance of women, the right side was operated on more frequently and the prevalence of overweight patients was high in this sample. The minimum follow-up time was 1 year and the maximum was 13 years. 

**Table 1.** Participant demographic data.

	**N = 19**
Age	65.5 years ± 13.1 SD (32 to 81 years)
Male	3 (15.8%)
Female	16 (84.2%)
Right knee	12 (63.2%)
Left knee	7 (36.8%)
Weight	77.8 kg ± 13.9 SD (62 to 110 kg)
Height	165 cm ± 10 SD (149 to 188 cm)
BMI	28.8 ± 5.2 SD (23.1 to 43)
Follow-up time	4.2 years ± 3.0 SD (1 to 13 years)

BMI: body mass index; SD: standard deviation.

Thirteen knees (68.4%) were classified as Krackow type 1. Six knees (31.6%) were classified as Krackow type 2. No knees were classified as Krackow type 3.

The preoperative FT angle ranged from 13 to 40 degrees of valgus deformity. The mean preoperative FT angle was 22.3 degrees ± 6.0 SD. The postoperative FT angle ranged from 1 to 13 degrees. The mean postoperative FT angle was 7.3 degrees ± 3.6 SD (p < 0.05; 95% CI 11.5-18.6).

Only two knees (10.5%) failed to reach full extension. One of them had a 10- degree deficit and the other a 30-degree deficit.

No participant presented varus knee thrust in the clinical gait examination.

There was no positive varus stress test in extension, but two knees (10.5%) showed mild positive varus stress at 30 degrees of flexion. Both cases were classified as Krackow type 1 preoperatively. These two participants had moderate pain (VAS = 6) and one of them had an extension limitation of 30 degrees.

Five participants (26.3%) reported some knee pain. The VAS scale for these patients ranged from 2 to 6 (4.6 ± 1.9 SD), on a 0-10 scale. Two participants had mild pain and three participants had moderate pain.

## DISCUSSION

The main finding of this study was the low incidence of adverse events related to sliding osteotomy of the lateral epicondyle, even without screw fixation. Major problems such as reoperations, early loosening, deformities and varus thrust were not observed.

 Since their pioneering work in 1979, Insall, Scott and Ranawat ^11^ have described the technique of soft tissue ligament balancing through serial release of the iliotibial band, lateral aspect of the capsule, LCL and popliteus tendon. As it is a laborious technique, many variations have been described, including sequential Whiteside releases, Keblish lateral approach, tibial tube osteotomy, sliding osteotomy of the lateral condyle and pie crusting intra-articular releases. ^1^^,^^8^^,^^12^^-^^15^

 There are few reports with case series of lateral epicondyle sliding osteotomies to correct knee valgus deformity. The first description was by Brilhault et al. in 2002, ^8^ who reported a series of 13 patients, always fixed with screws. The authors report that they achieved satisfactory stability and alignment. Mullaji and Shetty ^16^ presented a computerized navigation technique to improve positioning accuracy, but did not describe clinical results. 

 Recently, three new articles with case series were published on the subject. Scior et al. ^17^ described a cohort of patients operated on between June 2007 and May 2014. Ninety-eight patients were treated with 98 sliding osteotomies, all fixed with screws. All knees achieved satisfactory alignment. However, seven revisions (7.1%) were reported, three of which were procedure-related. In two there was dehiscence of the lateral joint capsule, while in one there was loosening of the epicondyle. 

 In the study by Li et al. ^18^ 25 knees were included between 2011 and 2017, with a mean follow-up of 3.3 years. Adequate stability and alignment were achieved and there was no revision surgery. Furthermore, all osteotomies were also fixed with screws. 

 In the third study, Raut, Matar and Singh ^19^ described a retrospective case series with 25 knees and a mean follow-up of 5 years. There was an improvement in the clinical scale of the Oxford Knee Score and there were no cases of revision. In this study, fixation of the lateral epicondyle was not performed, corroborating the findings of our study. 

This study has some limitations. Firstly, it is a retrospective study, with the potential for bias inherent in this type of design. As this is an uncommon surgery and complications are rare, it would be unfeasible to conduct a prospective study in a single center. Secondly, we had a small number of participants. Arthroplasties in severe valgus knees, which require advanced techniques to achieve ligament balance, end up being uncommon. Thirdly, we did not assess bony union at the osteotomy site. We believe that union is always achieved, either by bone or soft-tissue consolidation, as there is no postoperative lateral instability. Finally, the lack of a control group prevents conclusions about the effectiveness of the technique compared to other ways of achieving ligament balance. A new study, already ethically approved, will soon be carried out with gait laboratory analysis to compare patients who underwent TKA with valgus deformity, with and without lateral epicondyle osteotomy.

## CONCLUSION

The results obtained in this sample are compatible with those described in the literature and corroborate the hypothesis that lateral epicondyle sliding osteotomy is a safe and effective procedure for ligament balance of knee prostheses with valgus deformity. They also support the hypothesis that screw fixation is unnecessary.
